# Acute Pesticide Poisoning in Children: Hospital Review in Selected Hospitals of Tanzania

**DOI:** 10.1155/2017/4208405

**Published:** 2017-12-26

**Authors:** Elikana Lekei, Aiwerasia V. Ngowi, Leslie London

**Affiliations:** ^1^Tropical Pesticides Research Institute, P.O. Box 3024, Arusha, Tanzania; ^2^School of Public Health and Social Sciences, Department of Environmental and Occupational Health, Muhimbili University of Health and Allied Sciences (MUHAS), P.O. Box 65015, Dar es Salaam, Tanzania; ^3^School of Public Health & Family Medicine, Faculty of Health Sciences, University of Cape Town, Anzio Road, Observatory 7925, South Africa

## Abstract

**Background:**

Acute pesticide poisoning (APP) is a serious problem worldwide. Because the burden of childhood APP is unknown in Tanzania, this study describes the distribution, circumstances, and patterns of APP involving children under 18 years in Tanzania.

**Methodology:**

A 12-month prospective study was conducted in 10 Tanzanian healthcare facilities in 2006 using a data collection tool for surveillance.

**Results:**

Of 53 childhood poisoning cases identified, 56.6% were female. The most common poisoning circumstances were accidents (49.1%) and suicide (30.2%). The most vulnerable children were 16-17 years old (30.2%). Suicide was significantly more common in females (PRR females/males = 1.66; 95% CI = 1.03–2.68) and accidental cases were more common in children aged 10 years or younger. Suicide was concentrated in children over 10 years, comprising 53% of cases in this age group. Organophosphates (OPs), zinc phosphide, and endosulfan were common amongst reported poisoning agents. The annual APP incidence rate was 1.61/100,000.

**Conclusion:**

APP is common among children in this region of Tanzania. Prevention of suicide in older children should address mental health issues and control access to toxic pesticides. Prevention of accidents in younger children requires safer storage and hygiene measures. Diverse interventions are needed to reduce pesticide poisoning among children in Tanzania.

## 1. Introduction

Acute pesticide poisoning (APP) has been previously noted as a serious problem in Tanzania both for children and adults [1, 2] and in other developing [3–5] and developed countries [6]. The World Health Organization (WHO) reports that over 30% of the global burden of disease in children can be attributed to environmental factors, and that pesticides are a major contributor [7]. Evidence of APP in children is documented in various studies from South Korea [8], South Africa [9, 10], Canada [11], Turkey [12], and India [13]. The proportion of cases for which pesticides are responsible for childhood poisoning varied from about 35% in South Africa [9] to 47% in Canada in 2007 [11]. A survey of cases of children younger than 14 years seen in one year at a tertiary children's hospital in South Africa identified 311 poisoning cases arising from pesticides poisoning with 6 deaths [10].

Other adverse health effects arising from pesticide exposure in children have been reported. In a study conducted in Egypt, children aged 9–15 years, who work seasonally in cotton farms to apply pesticides, reported significantly more neurological symptoms and reduced cholinesterase levels compared to controls [14].

Currently, Tanzania has no surveillance system for acute pesticide poisoning amongst children. Poisoning is reported in the health management information system within Tanzanian hospitals with minimum information on the causative agent. Previous studies have reported the lack of specificity of reporting of poisoning in hospital information systems [15]. The lack of data on childhood APP is particularly a concern given the large burden of disease due to pesticides in children noted in less developed countries [16–18].

Children, particularly those under 5 years of age, can be exposed to pesticides directly through ingestion involving swallowing pesticides or their concentrates, consumption of contaminated food, or hand-to-mouth behaviors typical of early childhood development. A study from Peru reported that 24 children died after drinking powdered milk substitute contaminated with the organophosphate methyl parathion [19].

Dermal absorption can occur with exposure to contaminated clothing or to dust or residues on floors and other surfaces or objects. Use of agricultural pesticides to control domestic pests and vermin has resulted in accidental poisoning of young children in urban homes in South Africa [20]. In rural areas of South Africa, it has been reported that a cause of child pesticide poisoning is the unknowing use by farm workers of toxic pesticides to spray beds of household members to control bedbugs, fleas, and other pests [21].

Other possible sources for exposure in this age category include pesticide drift [22] and unattended empty pesticide containers [23]. When pregnant and lactating women have body burdens of pesticides, newborns and infants may be exposed to them through in-utero placental transfer. Children may also be exposed to pesticides through forms of hazardous child labour involving fieldwork spraying pesticides, washing their parents' contaminated work clothes, or reentering fields to work following spray application. Young children may also be exposed to pesticide by maternal work with it if they accompany parents to work. Children in rural areas may also have to walk through sprayed fields to reach school.

These indirect risks of childhood pesticide are documented in various studies worldwide. A Tanzanian study in 2005 reported that mothers who retail pesticides may take their children with them to their shops where pesticides are stocked and sold [23]. Studies in the United State (US) confirm that children of agricultural families are at risk of being exposed to agricultural pesticides even if they are not involved in farm activities [24] and that children living with parents who work with agricultural pesticides, or who live in proximity to pesticide-treated farmland, have higher exposures than other children living in the same community [25].

Children are more vulnerable than adults to the adverse health effects of pesticides [26–30] as a result of (i) their small body size resulting in a higher body surface area relative to body weight, (ii) differing metabolism of toxic chemicals, and (iii) the rapid growth and development of children's organ systems. Infants and children interact with their environment differently—such as learning through touch and hand-to-mouth behavior.

Inexperience, lack of maturity, illiteracy, and an inability to assess risk make accidental ingestion of pesticides amongst children more likely. Children have higher metabolic rates and their bodies are less able to detoxify and expel harmful chemicals. In short, children are absorbing a higher load of pesticides at a time when their bodies are still developing and are thus least equipped to protect themselves. Therefore, data on the extent of risk in children from pesticide exposure are needed to make preventive interventions to protect children from harmful effects of chemicals [31].

Because the magnitude of APP in children in Tanzania is unknown, this study was undertaken with the aim of estimating the burden of APP amongst children and characterizing the patterns of APP affecting children reported in healthcare facilities in Tanzania. Because Tanzania currently lacks a comprehensive surveillance system for acute pesticide poisoning, such a study would provide recommendations to address this gap.

## 2. Materials and Methods

### 2.1. Data Collection

Data on childhood APP were obtained as part of a hospital-based review in 4 selected districts of Tanzania where intensive coffee and vegetable production was associated with intense pesticide use. The study methods have been previously described [15] and involved a 12-month retrospective review of cases of pesticide poisoning presenting to 30 facilities in 2005 and a 12-month prospective study in 10 facilities in 2006. This paper reports on the findings involving children. Out of the 230 cases reported in the prospective study, 53 (23%) cases were children and the rest were adults [15].

These 10 facilities were selected as having recorded the majority of APP cases in the retrospective study in 2005 and included regional (*n* = 3), referral (*n* = 2), and district (*n* = 1) hospitals and a subset of health centers and dispensaries (*n* = 4) selected from the Arusha district near the Tropical Pesticide Research Institute (TPRI), in which the study was conducted. These smaller facilities closer to the TPRI were included because of logistic ease.

This study focuses on data reported for children in the prospective study in 2006. Since children are legally defined in Tanzania (and in many other countries) as persons under 18 years, we used 18 years as the cut-off to define children in this study. The prospective study involved intensive training of facility staff to record all APP cases with the aim of improving quality of data collection. Comparison of retrospective review to prospective data collection suggested that the training intervention was successful in reducing missing information on the circumstances, outcomes, and agents responsible for APP by about 50%, 50%, and 20%, respectively [15].

The data collected were, firstly, retrieved from the register book at the facility and included patient registration number, date of consultation, location, gender, circumstances of poisoning, and outcome. Secondly, the patient registration number was used to locate the patient folder from which further information was extracted including agents responsible, circumstances, and treatment of poisoning. Comparisons were also made to data found in the poisoning register. The data were collected using a standardized data collection sheet by specifically trained medical data recorders.

A case of APP was defined in this study as a diagnosis of APP made by the clinician and recorded in either the register or patient folder or both. In general, clinician diagnosis was based on a history of exposure (from the patient, relative, or accompanying person) to one or more pesticides and clinical manifestations of poisoning or specific laboratory test results compatible with APP, within 14 days of exposure. Children were defined as persons aged 17 years or younger.

### 2.2. Data Analysis

Description of childhood poisoning by age was done using 5-year intervals. The group of children under 6 is a common category used internationally and the grouping of children 6 to 17 enables comparison to similar studies conducted elsewhere. Poisoning circumstances were classified as (a) suicide, (b) accidental, (c) occupational, (d) homicide, or (e) unknown, based on information recorded in the diagnostic sections of the patient folders or directly documented in the patient register books. In cases where the two sources disagreed, information from the patient folder was used, because information completed by a medical professional was judged to be more likely precise as compared to the register book which was completed by more junior staff.

The outcomes of APP were classified as (a) recovery, (b) absconded, (c) referred, (d) residual disability after discharge, (f) death, or (g) unknown. A second pair of analyses of outcomes further reduced classification as (a) fatal versus nonfatal and (b) known versus unknown outcomes. Agents responsible for APP were classified as (a) specific (active ingredient was identified), (b) nonspecific (active ingredient was known by general category), or (c) unknown (active ingredient was not known to the clinician and/or not recorded). Comparisons involving circumstances and outcome of poisoning, agents, gender, and age were conducted using *χ*^2^ tests for cross-tabulated categorical data and *t*-tests for continuous data such as age. We used different cut-offs at 5 and at 10 years to identify older children compared to younger children so as to generate sufficient numbers in each group. The strength of associations was estimated in terms of prevalence risk ratios (PRR) with 95% confidence intervals. The analyses were conducted using SPSS statistical package version 16.0 and STATA statistical package version 10.0 [32, 33].

Data on APP cases were used as numerator data to calculate morbidity rates stratified for gender, geographical area, and age. To calculate denominators for rates, population census data was obtained from the Tanzania Bureau of Statistics based on a national census conducted in 2002 [34] and population for 2007 adjusted for annual population growth of 2.7% [35].

### 2.3. Ethical Considerations

Because the study involved record review and no data were collected directly from individuals, there was no consent required. To ensure confidentiality, patient names were replaced by codes which were used as identifiers in data analysis. Ethical approval was secured from the TPRI, the National Institute for Medical Research (NIMR) in Tanzania (Ref. NIMR/HQ/Vol XI/371), and the University of Cape Town (Ref. 328/2004).

## 3. Results

Of the 10 facilities followed up for 12 months during the study, 9 facilities reported 53 cases of childhood APP. The facility which did not report children poisoning was a small health center near Arusha. The highest number of cases (*n* = 22; 42%) was reported from the regional hospital. The 53 cases comprised 23% of the total of 230 cases (children and adults) recorded in the course of 2006 [15].

### 3.1. Characteristics of Cases Reported

#### 3.1.1. Age Category and Gender (*n* = 53 Cases)

The age category with highest proportion of poisoned children was 16-17 years (30.2%) (Table 1). Of the 53 poisoned children, 23 (43.4%) were males and 30 (56.6%) females. Female APP cases were significantly older than male cases (mean ages: 12.3 years versus 9.1 years, respectively; *p* = 0.03).

#### 3.1.2. Circumstances of Poisoning

The two most commonly recorded circumstances of poisoning were accidents (*n* = 26; 49.1%) and suicide (*n* = 16; 30.2%). Occupational APP was reported in 4 cases (Table 1). For males, the most common circumstance was accidental poisoning (65% of all male APP), while for females, suicide was the most common (40% of all female APP). Three of the four occupational cases of APP in children occurred in females. Lack of data on circumstances of poisoning was low in both male (9%) and female (13%) cases.

The proportion of circumstances due to suicide was significantly higher in females compared to males (46.2% versus 19.0%; PRR females/males = 1.7; 95% CI = 1.0–2.7). Suicide was concentrated entirely in children older than 10 years, comprising just over half of all cases (16 out of 30 cases) in this age group but was not present at all in younger children (*p* < 0.005). Cases of suicide were significantly older than accidental cases (mean age 15.7 years versus 6.5 years, respectively; *p* < 0.005). Accidental cases were significantly more common in children aged 10 years or younger (100%) than in children older than 10 Years (10%) (*p* < 0.005).

Only two children were reported to have died (3.8%). However, the outcome was unknown for 10 children (18.9%).

#### 3.1.3. Poisoning Agents

The agents responsible for poisoning are reported in Table 2. In over half of the cases, (50.9%) the pesticide involved was unknown. Organophosphates were the most commonly reported specific agent, accounting for 7 of the 11 cases where specific agents could be identified. All OPs reported were WHO Class II toxins. Two cases of poisoning were reported with zinc phosphide which is a WHO Class Ib toxin. Nonspecific products such as livestock dip (1.9%), food poisoning agents (food items contaminated with unknown pesticides) (20.8%), and rat poisoning agents (5.7%) accounted for 28.2% of all products reported.

The majority of the specific agents reported (*n* = 19; 90.9%) were WHO Class I and II pesticides and OPs accounted for 63.6% of all known poisoning agents (Table 2).

The proportion of circumstances due to suicide was higher in cases with unknown agents compared to cases with known agents but the difference was not statistically significant (40.0% versus 29.6%; PRR unknown/known = 1.3; 95% CI = 0.7–2.5). The proportion of known agents was significantly higher in younger (age: 0–5 years) compared to older (age: >5 years) children (62.2 versus 25.0%; PRR younger/older = 1.6; 95% CI = 1.1–2.3). The proportion of cases for which the outcome was known was higher for females compared to males (PRR 1.4; 95% CI = 0.8–2.1), for older compared to younger children (PRR = 1.4; 95% CI = 0.6–3.5) and for suicidal circumstances compared to other circumstances (PRR = 1.6; 95% CI = 0.8–2.7), but none of these differences were statistically significant.

### 3.2. Children Poisoning Rates

The annual IR for APP was 1.61/100,000 with higher rates reported for the Arusha region. The age group 16-17 years reported the highest IR (6.17/100,000) (Table 3). Because there were only 2 fatal cases, incidence rates for mortality were not calculated.

## 4. Discussion

This study identified 53 acute pesticide poisoning cases in one year involving children in 9 of the 10 facilities in this study. The fact that all but one facility reported a childhood poisoning case indicates that childhood pesticide poisoning is ubiquitous in the study area. With intensive surveillance and improvements to the reporting system, a larger number of cases are likely to be captured in the health management information system.

The regional hospital reported the highest number of cases (*n* = 22). As a regional hospital, it would be expected to draw more serious poisoning cases through referral. This could also be partly explained by better recording in regional compared to other facilities. Additionally, it could also be the result of widespread availability of toxic pesticides such as cholinesterase inhibitors like chlorpyrifos, used heavily in the surrounding Kilimanjaro area for coffee bean cultivation.

The age distribution of APP in children suggests the highest proportion of APP is located in the age group 16-17 years and lowest in the age category 1–5 years. This may be explained by the fact that younger children under five receive more parental attention than adolescents whereas older children 16 to 17 years may be at higher risk of suicide (given psychological challenges faced by young people approaching adulthood) and face occupational hazards under conditions of child labour.

Children under 5 years comprised 21% of all cases of poisoning of children in the study. This proportion was lower than reported in South Africa (34.4%) [9], Taiwan (35.4%) [36], USA (57%) [37], and Brazil (41%) [38]. The difference may be attributable to poorer reporting in Tanzania as compared to USA, South Africa, Taiwan, and Brazil.

In terms of gender, this study found that the majority of childhood poisoning cases were female. These findings are consistent with studies in India [39] and Nepal [40] but in contrast to findings from studies in the USA [37], South Africa [9], and Nicaragua [41] which report a male predominance in poisoned children. For occupational poisoning, the reason for this trend could be the nature of pesticide handling tasks which are traditionally conducted by males in different countries. In USA, South Africa, and Nicaragua it is likely that males conduct handling tasks like mixing pesticides and field spraying while females do simple tasks like storage maintenance and harvesting. However, the relevance of findings amongst adult farm workers remains hypothetical for children since there has been insufficient research into gender differences amongst children handling pesticides in the region.

Suicide was concentrated exclusive amongst older children, particularly females. This gender predilection for suicide is consistent with findings in the literature from Sri Lanka, China, Chile, and Korea [42–46]. Preventive measures should address the mental health issues facing young people, as well as limiting easy access to toxic pesticides for young people facing emotional distress. The Sri Lankan experience of banning the most toxic pesticides which reduced mortality from suicide in the country [47] may be relevant for Tanzania, given that most of the agents which could be identified in this series involved more toxic agents classified as WHO Class I and II pesticides. This is further supported by findings of a recent systematic review that found that national bans on highly hazardous pesticides, rather than other forms of sales restrictions, seem to be effective in reducing pesticide-specific and overall suicide rates [48].

Accidents were the single highest category of circumstance (49.1%) and were more common in males (65.2%). The findings in this study are similar to studies conducted in South Africa, which reported that the main circumstance of children exposure to pesticides was accidental ingestion of organophosphates (OPs), either as residues on unwashed fruit or from poorly marked storage containers, or from dermal and respiratory absorption following OP application for pest control in and around homes [49].

Similarly, a study conducted in Zambia reported that the major circumstances of poisoning for children poisoning were accidental [50]. Prevention of accidental poisoning in children may require safer storage, improved handling, better hygienic practices, and greater awareness in rural communities, although a recent randomized controlled trial of safer storage methods appeared to show no benefit in reduced APP or suicides [51]. Legislative measures to restrict the most toxic pesticides may therefore be more useful for low and medium income countries (LMICs) [48].

Occupational circumstance was uncommon, accounting for only 7.5% of APP cases. Nevertheless, the presence of four APP cases in working children is an indication that children are involved in pesticides application, which is against the Tanzania Law of the Child Act of 2009 [52] and Regulations of 2012 [53]. Hazardous work prohibited in agriculture is outlined in the Tanzanian Regulations for Child Employment of 2012 and includes, among other activities, “application of pesticides and fertilizers” [53]. This is an indication of the ongoing practice of child labour and signals the need for better enforcement of regulations prohibiting hazardous child labour under the International Programme to Eliminate the Worst Forms of Child labour (IPEC).

This situation is similar to findings of a Philippine study which found that children start working in vegetable farms with pesticides as early as 6 to 9 years old [54]. A study in South Africa reports involvement of children in the sale and distribution of pesticides in street markets [55]. A study conducted in India reports that child labour in agriculture sector accounts for 80% of child labourers in India and 70% of working children globally [56].

There were only 2 fatalities (3.8%) reported amongst the children cases, which is about 40% lower than the case fatality rate (CFR) found in adults [15]. Though this difference was not statistically significant (*p* = 0.3), this difference, given small number and low power, may still suggest possible better care for children in healthcare facilities, or that poisonings among children are less severe.

However, the outcomes for 10 cases were unknown and these might have included unrecorded fatal outcomes. Where there were fatal outcomes, delays in accessing timely care may have contributed to the deaths.

The poor percentage of cases where poisoning agents could be identified (about half of the cases were of unknown agents) could reflect the fact that children have little capacity to identify or remember agents in cases of poisoning and that caregivers are not present when the poisoning occurs so they cannot identify the agent.

Organophosphates (OPs) emerged as the single most important group of agents responsible for poisoning (*n* = 7 or 14%) and this is consistent with studies globally. In Tanzania, for example, OPs are only 16% of all registered pesticides [57]. This implies that their toxicity plays a major role in the involvement of these products in poisoning. In a study conducted in South Africa, the commonest groups of poisoning agents were 203 cholinergic (including organophosphates and carbamates) [20]. In another study by Sungur and Güven [58] thirty-two (68%) of patients were reported to be poisoned by organophosphates. However, given the large number of APPs with unknown agents, it is possible that the true proportion of involving OPs was much higher. Regulatory action to control the most hazardous pesticides such as OPs is therefore warranted.

The study reported cases of poisoning involving endosulfan and zinc phosphide. Endosulfan is a toxic pesticide earmarked for elimination under the Stockholm Convention on Persistent Organic Pollutants (POPs) and is also listed under the Rotterdam Convention, although its inclusion was stalled for many years because of the seeming absence of data on severe incidents involving endosulfan [59]. It has been banned for health reasons in Tanzania since 2013. The decision to list a pesticide, such as endosulfan, under the Rotterdam Convention requires notification of severe adverse events. The absence of a surveillance system that identifies such poisonings means that the information base on which decisions under the convention are made is faulty. This illustrates the importance of good surveillance informing global pesticide policy.

Zinc phosphide is an extremely toxic agent that is not registered in Tanzania. Illegal distribution of this product for rat control in households is a potential reason for the association with childhood poisoning. The product is thought to be imported illegally from neighboring countries and its presence in Tanzania as a cause of APP in children points to the need for strengthening border controls by the government to discourage this practice.

The study estimated the first population-based incidence rates for pesticide poisoning for children in northern Tanzania. The childhood IR for APP in this study (1.61/100,000) was lower than rates found in studies of APP in children in Korea (3.6/100000) [60] and Central America, (5.7/100000) [61] but higher than rates in USA (0.7/100000) [62]. The difference may arise because Korea and Central America have surveillance systems in place for pesticide poisoning. There may also be differences in the toxicity of agents to which children of Central America and Korea are exposed. Conversely, lower rates in the USA (0.7/100000) may be attributable to stronger occupational and environmental safety regulations and enforcement in terms of safety consideration to pesticide users.

## 5. Conclusion

The study indicates that acute pesticide poisoning is common among children in northern Tanzania with estimated IR of 1.61/100,000. The most common known agents were WHO Class I and II pesticides including a number of organophosphates. The most common circumstances of APP in children were accidents for children 10 years or younger and suicide for children over 10. To reduce APP related to accidents and suicide, attention should be paid to safer storage, improved hygiene measures, and control of access to toxic pesticides. Occupational APP, though uncommon, signals the need for attention to the eradication of worst forms of child labour. Multifaceted intervention efforts are needed to reduce pesticide poisoning among children in Tanzania.

## Figures and Tables

**Table 1:** Age category and circumstances of poisoning for APP amongst children in Tanzania.

Age category	Known	Unknown	Accidental	Occupational	Suicide	Homicide	Total
1–5	11	0	11	0	0	0	11
6–10	12	0	12	0	0	0	12
11–15	11	3	2	2	6	1	14
16-17	13	3	1	2	10	0	16
Male	21	2	15	1	4	1	23
Female	26	4	11	3	12	9	30
Total	47	6	26	4	16	1	53

**Table 2:** Classification of agents responsible for APP in children in Tanzania.

Product	Chemical group	WHO Class	Frequency	Percentage by category	Percentage of all agents
*(1) Known products*					
Zinc Phosphide	IN	1b	2	18.2%	3.7%
OP	OP	II	7	63.6%	13.2%
Sulphur	IN	IV	1	9.1%	1.9%
Endosulfan	OC	II	1	9.1%	1.9%
*S total 1*			*11*	*100.0%*	*20.7%*
*(2) Unspecific products*					
Livestock dip	UN	UN	1	6.67%	1.9%
Food poisoning	UN	UN	11	73.33%	20.7%
Rat poison	UN	UN	3	20.00%	5.6%
*S total 2*			*15*	*100.00%*	*28.2%*
*(3) Unknown*					
UN			27	100.00%	50.9%
*S total 3*			*27*	*100.00%*	

IN: inorganic; OP: organophosphate; OC: organochlorine; UN: unknown.

**Table 3:** Incidence rate, mortality rate, and case fatality rate for APP for children in Tanzania.

	Poisoning cases per year	Population	Annual IR (per 100,000)
Regions			
Arusha	15	738,618	2.03
Mwanza	25	1,764,238	1.42
Kilimanjaro	13	782,442	1.66
*Total*	*53*	*3,285,298*	*1.61*

Age groups			
1 to 5	11	1,258,717	0.87
6 to 10	12	976,393	1.23
11 to 15	14	790,902	1.77
16 to 17	16	259,286	6.17
*Total*	*53*	*3,285,298*	*1.61*

Gender			
Males	23	1,649,495	1.39
Females	30	1,635,803	1.83
*Total*	*53*	*3,285,298*	*1.61*
